# Tissue-Engineered Vascular Graft with Co-Culture of Smooth Muscle Cells and Human Endothelial Vein Cells on an Electrospun Poly(lactic-co-glycolic acid) Microtube Array Membrane

**DOI:** 10.3390/membranes11100732

**Published:** 2021-09-27

**Authors:** Chee Ho Chew, Bo-Long Sheu, Amanda Chen, Wan-Ting Huang, Tsai-Mu Cheng, Chun-Ming Shih, Austin Chang, Chien-Chung Chen

**Affiliations:** 1 Graduate Institute of Biomedical Materials & Tissue Engineering, College of Biomedical Engineering, Taipei Medical University, Taipei 11052, Taiwan; chchew88@gmail.com (C.H.C.); levis19930507@gmail.com (B.-L.S.); sandyhuang@mtamtech.com (W.-T.H.); 2 Department of Biochemistry, University of Washington, Seattle, WA 98195, USA; mandycookie13@gmail.com; 3 The Ph.D. Program for Translational Medicine, Taipei Medical University, Taipei 11052, Taiwan; tmcheng@tmu.edu.tw; 4 Graduate Institute of Biomedical Informatics, College of Medical Science and Technology, Taipei Medical University, Taipei 11052, Taiwan; 5 Taipei Heart Institute (THI), Taipei Medical University, Taipei 11052, Taiwan; cmshih53@tmu.edu.tw; 6 Division of Cardiology, Department of Internal Medicine, Taipei Medical University Hospital, Taipei 11052, Taiwan; 7 Graduate Institute of Clinical Medicine, College of Medicine, Taipei Medical University, Taipei 11052, Taiwan; 8 Core Facility Center, Taipei Medical University, Taipei 11052, Taiwan; austinc99@tmu.edu.tw; 9 International Ph.D. Program in Biomedical Engineering, College of Biomedical Engineering, Taipei Medical University, Taipei 110, Taiwan; 10 International Ph.D. Program for Cell Therapy and Regeneration Medicine, College of Medicine, Taipei Medical University, Taipei 110, Taiwan; 11 Ph.D. Program in Biotechnology Research and Development, College of Pharmacy, Taipei Medical University, Taipei 110, Taiwan

**Keywords:** Poly(lactic-co-glycolic acid) (PLGA), Microtube Array Membrane (MTAM), electrospinning, Tissue Engineered Vascular Grafts (TEVG), Smooth Muscle Cells (SMCs), Human Endothelial Vein Cells (HUVECs)

## Abstract

Coronary artery disease is one of the major diseases that plagues today’s modern society. Conventional treatments utilize synthetic vascular grafts such as Dacron^®^ and Teflon^®^ in bypass graft surgery. Despite the wide adaptation, these synthetic grafts are often plagued with weaknesses such as low hemocompatibility, thrombosis, intimal hyperplasia, and risks of graft infection. More importantly, these synthetic grafts are not available at diameters of less than 6 mm. In view of these challenges, we strived to develop and adapt the electrospun Poly Lactic-co-Glycolic Acid (PLGA) Microtube Array Membrane (MTAM) vascular graft for applications smaller than 6 mm in diameter. Homogenously porous PLGA MTAMs were successfully electrospun at 5.5–8.5 kV under ambient conditions. Mechanically, the PLGA MTAMs registered a maximum tensile strength of 5.57 ± 0.85 MPa and Young’s modulus value of 1.134 ± 0.01 MPa; while MTT assay revealed that seven-day Smooth Muscle Cells (SMCs) and Human Umbilical Vein Endothelial Cells (HUVECs) registered a 6 times and 2.4 times higher cell viability when cultured in a co-culture setting in medium containing α-1 haptaglobulin. When rolled into a vascular graft, the PLGA MTAMs registered an overall degradation of 82% after 60 days of cell co-culture. After eight weeks of culturing, immunohistochemistry staining revealed the formation of a monolayer of HUVECs with tight junctions on the surface of the PLGA MTAM, and as for the SMCs housed within the lumens of the PLGA MTAMs, a monolayer with high degree of orientation was observed. The PLGA MTAM registered a burst pressure of 1092.2 ± 175.3 mmHg, which was sufficient for applications such as small diameter blood vessels. Potentially, the PLGA MTAM could be used as a suitable substrate for vascular engineering.

## 1. Introduction

In this modern era, the sedentary life style has significantly contributed to a myriad of ‘diseases of the modern life’. One of the leading diseases is Coronary Artery Disease (CAD), which is the result of the narrowing of the blood vessels that ultimately leads to the blockage of it. Consequently, the tissues/organs that are reliant on these blood vessels for gases, nutrients, and waste exchange die [1]. Globally, CAD-related deaths is projected to reach a staggering 23 million by the year 2030 [2].

Currently, one of the most commonly performed surgeries for the treatment of CAD is Coronary Artery Bypass Graft (CABG). Vascular sources for CABGs are generally sourced from either autologous or synthetic sources. However, to date, vascular sourced from autologous sources generally produces the best outcome [3]. Despite these advantages, it is common to find vascular of poor quality. For instance, the saphenous vein is one of the major donors of autologous sourced vascular grafts, but up to 30% of these veins are unusable due to poor quality [4].

For the past 30 years, significant efforts have been made to develop synthetic vascular grafts. Several commercially available options such as the Dacron^®^ and Teflon^®^ have been widely used clinically. These successes are largely limited to large and medium diameter grafts (>6 mm), while largely unsuccessful at a smaller diameter. For instance, some polytetrafluoroethylene (PTFE) based synthetic grafts only retained a patency value of 32% while their larger diameter counterparts maintained a patency value of 90% even after a decade [1,5,6]. Additionally, the failure of these synthetic grafts have been largely associated with thrombosis, which is the result of a missing functioning endothelium [7]; intimal hyperplasia, which is caused by mismatch between native and synthetic grafts, diameter mismatch, damage to the graft, flow disturbance, stresses exerted by suture lines, and the lack of a functioning endothelium [8,9,10,11,12,13].

Recently, there have been significant efforts to develop tissue engineered vascular grafts (TEVGs) [4,14,15]. Generally, there are several requirements for TEVGs such as possessing a surface to enable it to resist thrombosis, non-immunogenic, mimic the mechanical strength of native blood vessels, and resistant to infections while being fully biocompatible [16,17,18,19]. Additionally, it should also be fully compliant with the implant site to prevent any mismatch/stresses around the anastomosis, which could potentially disrupt the flow dynamics [12,20,21]. Ultimately, the TEVGs should be able to grow, self-repair, and fully assimilate with the connecting native blood vessels [1].

Currently, the most widely conducted clinical trials of TEGVs are the scaffold-based technique, which utilizes synthetic scaffold mesh [1]. In an example, Hibino et al. worked on highly porous degradable polyglycolide (PGA) reinforced, poly-L-lactide (PLLA) and poly-ε-caprolactone (PCL) co-polymer based vascular grafts that functioned as extra cardiac cavopulmoary conduits for the treatment of single ventricular physiology. These scaffolds were seeded with autologously derived bone marrow-derived mononuclear cells (BM-MNCs) and the seven-year study revealed fully degraded scaffolds with no aneurysm, rupture, or ectopic calcification while being infection free. Most importantly, recognizable SMCs and an endothelium layer can be observed; and as a result, up to 40% of patients do not require any anti-coagulation therapy [1,22,23]. Further examples of success can be found in the use of polyglycolide (PGA) mesh seeded with porcine/human SMCs and endothelial cells (ECs) [3,24] and; PGA coated with polyhydroxybutyrate (P4HB) seeded with autologous ovine ECs and fibroblasts [25]. On the other hand, synthetic scaffold mesh that were directly implanted without any prior cell seeding was also successfully conducted by using synthetic scaffolds such as electrospun poly(glycerol sebacate) (PGS) and PCL [26] and woven PGA and PLLA [27].

With the advent of 3D printing, self-assembled TEVGs have been the recent focus of various research groups. These TEVGs were pioneered by cell sheet engineering and recent developments have included micro tissue aggregation and 3D cell printing [1]. Cell sheet-based TEVGs involved the wrapping of a layer of autologous SMCs followed by fibroblast around a tubular template. This self-assembled TEGV was then incubated in a perfusion bioreactor by up to eight weeks to allow for the individual cell layers to fuse together, resulting in a mechanically relevant TEVG capable of sustaining burst pressure of 2594  ±  501 mmHg, which is superior to that of native saphenous veins (SVs). Human trials revealed 20 months patency (4 in 10), which was in line with current clinical targets [15,18]. Subsequent developments such as micro tissue aggregate TEGVs was demonstrated in the use human artery fibroblasts and HUVECs into a tubular-shaped structure with extra-cellular matrix (ECM) acting as the binding agent and subsequently culture in a dynamic culture. Fourteen-day culture revealed significantly higher levels of ECM formation with blood vessel like tissues fully observable [28,29]. 3D printing self-assembled TEGVs were also successfully demonstrated in work by Forgacs et al., where human umbilical cord SMCs and dermal fibroblasts were 3D printed, resulting in branched vessel formation and high cell density tubular structure. Critically, this TEGV was capable of sustaining a burst pressure of 773 mmHg [30,31].

Despite these developments, several key issues remain unresolved and among them include the lack of grafts with small diameter with good patency, despite being used successfully at larger diameters; or plagued with several key issues such as compliance, thrombosis, and infection [5,6,32,33]. Furthermore, to date, there are no FDA-approved vascular grafts of less than 5 mm due to high chances of graft failure, which is commonly associated with thrombosis [34,35,36].

In view of these challenges, we strive to demonstrate the recently developed novel electrospun microtube array membrane (MTAM) as a potential vascular graft. Microscopically, MTAMs consists of several unique characteristics, which could prove to be highly beneficial vascular grafts. Among them include the high porous, ultra-thin lumen wall [37], which is extremely beneficial for co-cultures [38]; one-to-one connected fibers with high degree of alignment, which is extremely critical to induce directional proliferation of cells such as SMCs [39]; mechanically sound, capable of sustaining a maximum tensile strength of 1.44 ± 0.87 MPa, while retaining good flexibility, as indicated with a Young’s Modulus reading of 1.55 ± 0.26 MPa [37,40,41]; and being easily manipulated, allowing it to be rolled into a tubular form. In this study, we strive to demonstrate the MTAMs as a novel new substrate that is capable of being utilized as vascular grafts.

## 2. Materials and Methods

### 2.1. Co-Axial Electrospun PLGA MTAMs

18 wt.% of PLGA shell solution was prepared by dissolving PLGA (Green Square Materials, Inc, Taipei, Taiwan; LA/GA ratio: 75:25) in a co-solvent of dichloromethane (DCM, Mallinckrodt, Surrey, UK)/N,N-dimethylformamide (DMF, Tedia, Fairfield, OH, USA) at a ratio of 8:2. The core solution was prepared by dissolving 10 wt.% of polyethylene oxide (PEO, 900 kDa; Sigma-Aldrich, St. Louis, MO, USA) and polyethylene glycol (PEG, 35 kDa; Sigma-Aldrich, St. Louis, MO, USA) in 40 mL of double-distilled water (ddH_2_O). Both solutions were successfully electrospun at 5.5–8.5 kV, at a flow rate of 5 mL/h. (shell solution) and 4 mL/h. (core solution) on a rotating drum, which revolved at 200 rounds per minute (rpm) under ambient condition. The resulting PLGA MTAMs were washed with ddH_2_O overnight to remove the porogen and air-dried for 24 h.

### 2.2. Microstructure Analysis of Electrospun PLGA MTAMs

PLGA MTAMs were cut into desired dimensions and sputter-coated for 180 s (IB-2 coater, Hitachi, Tokyo, Japan). The sputter-coated PLGA MTAMs were analyzed with a scanning electron microscope (SEM; S-2400, Hitachi, Tokyo, Japan) using an accelerating voltage of 15 kV, and the microstructures of the PLGA MTAMs were analyzed with an image analysis software (Image J, National Institutes of Health, Stapleton, NY, USA).

### 2.3. Mechanical Properties of Electrospun PLGA MTAMs

Defect-free electrospun PLGA MTAMs were selected and cut into rectangles with dimensions of 1 cm × 2.5 cm (width × length). The respective PLGA MTAMs were mounted onto the mounting of a LF Plus testing machine (LLYOD Company, Barking, London, UK) parallel to the direction of pull. The resulting stress-strain curve was recorded and analyzed to determine the maximum tensile strength and Young’s modulus of the electrospun PLGA MTAMs (extension rate: 120 µm/s).

### 2.4. Plasma Treatment (Acetic Acid; AA)

The AA plasma device utilized in this process was developed internally by Prof. Ko-Shao, Chen (Datong University, Taipei, Taiwan). Prior to any cell culture experiments, the respective electrospun PLGA MTAMs were cut into desired dimensions and treated with AA plasma (power: 10 watts; pressure: 100 mTorr) for 5 min. Next, the AA plasma treated MTAMs were soaked in 5% gelatin solution (Sigma-Aldrich, St. Louis, MO, USA) for 30 min and air-dried in a lamina flow under UV radiation for 30 min.

### 2.5. Contact Angle Measurement

PLGA MTAMs were cut into the desired dimensions and carefully adhered onto a glass slide using double sided tape. Next, the glass slide was mounted onto the platform of the angle goniometer (Digi drop PROD, GBX, Dublin, Ireland) and 5 µL of ddH_2_O was dispensed to form a small droplet at the tip of the dispensing needle. The automated measuring process was triggered and the respective contact angles were determined from the pictures of the water droplet on the electrospun PLGA MTAMs.

### 2.6. Liquid–Liquid Porometry

The electrospun PLGA MTAMs were cut into dimensions of 1 cm × 6 cm. Next, one end of the PLGA MTAMs were sealed off with epoxy sealant (Slink, Taipei, Taiwan); and stack in sets of 10 layers and attached to the porometry adapter with generous amount of epoxy sealant (Slink, Taipei, Taiwan). The entire set was mounted into the porometry with isopropyl alcohol (PMI, Ithaca, NY, USA) as the wetting media. The automated measuring process was triggered and the respective data was collected.

### 2.7. SMCs, HUVECs (Cell Culture), and Haptoglobin α-1

SMCs, HUVECs, and Haptoglobin α-1 were generously provided by Prof. Tsai-Mu Cheng of the Ph. D. Program for Translational Medicine (Taipei Medical University, Taipei, Taiwan). SMCs were cultured in 10% fetal bovine serum (FBS, Gibco, Waltham, MA, USA) in HEPES-buffered Dulbecco’s modified Eagle’s medium (HDMEM, Gibco, Waltham, MA, USA), while HUVECs were cultured in 5% FBS EBM-2 (Lonza, Basel, Switzerland). All culture mediums contained 50 U/mL of Penicillin and 50 mg/mL of Streptomycin (P/S; Gibco, Waltham, MA, USA). Haptoglobin α-1 was added to the respective mediums at a concentration of 0.2 mg/mL, as required. The respective cell cultures were incubated at 37 °C in a 5% CO_2_ atmosphere in an incubator (SCA-165DS, Astec, Fukuoka, Japan). Upon achieving 80% confluence, the respective cells were sub-cultured.

Cell cultures with PLGA MTAM as the graft entailed the siphoning of 1 × 10^4^ per μL of SMCs cell suspension into a pre-sterilized electrospun PLGA MTAMs of 0.5 cm × 2.5 cm (width × length). The respective ends of the electrospun PLGA MTAMs was folded over to ensure a tight seal. 1 × 10^5^ per μL of HUVECs were seeded on the exterior of the electrospun PLGA MTAMs and the entire cell seeded PLGA MTAM was transferred into a 12-well plate (ThermoFisher, Waltham, MA, USA) containing a medium combination of HDMEM and EBM-2 at a ratio of 3:7. Haptoglobin α-1 was added to the respective mediums at a concentration of 0.2 mg/mL, as required. All cell cultures were incubated at 37 °C in a 5% CO_2_ atmosphere in an incubator.

### 2.8. 3-(4,5-Dimethylthiazol-2-yl)-2,5-diphenyltetrazolium Bromide (MTT) Assay

At the pre-determined time of day 1, 4, and 7; samples were retrieved and the remaining medium discarded. Next, the samples were returned to their respective wells and 0.5 mg/mL MTT solution (Sigma-Aldrich, St. Louis, MO, USA) was added to each well and incubated for 180 min at 37 °C in a 5% CO_2_ atmosphere in an incubator. This was followed by the addition of dimethyl sulfoxide (DMSO; Sigma-Aldrich, St. Louis, MO, USA) and the corresponding absorbance was measured at 570 nm using an ELISA spectrophotometer (TECAN Sunrise ELISA reader, TECAN, Männedorf, Switzerland) to determine the optical density (OD) value.

### 2.9. Assembly of TEVGs

Pre-treated (AA plasma & gelatin coated) electrospun PLGA MTAMs were cut into dimension of 1 cm × 4 cm. Next, 1 × 10^4^ cells per μL of SMCs were siphoned into the lumens of the PLGA MTAMs and the respective ends were folded over to form a tight seal. The cell-loaded PLGA MTAM was rolled around a plastic pipette that had a diameter of 4 mm (the direction of roll was perpendicular to the length of the plastic pipette), which acted as the template. The meeting edges were secured with Histoacryl^®^ surgical glue (B-Braun, Barcelona, Spain) and allowed to set, as per the manufacturer’s instructions. Next, 2 × 10^5^ per μL of HUVECs were seeded onto the surface of the inner diameter of the tubular structure and incubated in medium (HDMEM:EBM-2 = 3:7; and 0.2% of haptoglobin α-1) at 37 °C in 5% CO_2_ between 1–8 weeks. At the pre-determined time, the respective samples were retrieved for imaging and histological examination.

### 2.10. Immunohistochemistry Staining

At the pre-determined time, samples were retrieved and fixed with 4% paraformaldehyde (PFA; Sigma-Aldrich, St. Louis, MO, USA) and rinsed with phosphate buffer saline (PBS; Sigma-Aldrich, St. Louis, MO, USA). This was followed by a 0.5% Triton X-100 (Bionovas, Toronto, ON, Canada) immersion, PBS washing, and a final immersion in a 3–5% bovine serum albumins (BSA; Sigma-Aldrich, St. Louis, MO, USA). Next, the SMCs were stained with a 150 fold α-SMA (Sigma-Aldrich, St. Louis, MO, USA), while the HUVECs were stained with a 50 fold CD 31 (Millipore, St. Louis, MO, USA) for 24 h at 4 °C. After 24 h, excess primary antibodies were rinsed off with PBS and; 200-fold anti-mouse IgG (H+L; Sigma-Aldrich, St. Louis, MO, USA) and anti-mouse IgG was added to the respective samples. The respective samples were left in dark conditions for 1 h, after which, a 5000 fold DAPI solution (Abcam, Cambridge, UK) was added. Lastly, the samples were observed with the following imaging equipment namely, fluorescence microscope (Leica, Wetzlar, Germany), laser confocal microscope (Leica, Wetzlar, Germany), and deconvolution fluorescence image microscope (GE Healthcare, Chicago, IL, USA).

### 2.11. Gel Permeation Chromatography (GPC)

Vascular graft samples of the PLGA MTAMs with similar dimensions were pre-cut and washed with double-distilled water (ddH_2_O) and dried in a desiccator set at room temperature for 24 h. Once dried, the samples were dissolved in tetrahydrofuran (THF; Sigma-Aldrich, St. Louis, MO, USA) and analyzed with GPC.

### 2.12. Burst Test of TEVGs

Pre-determined samples of TEVGs were retrieved and transferred into PBS. While being immersed in PBS, the leading end was connected to a three-way connector, where one end was connected to a pressure gauge (Tiren, Taipei, Taiwan) and the other end to the TEVG. Next, both ends were attached to rubber tubing, and sealed with silicone sealant (Dow Corning, Midland, MI, USA) to prevent leaks. Next, the rubber tube connected to the leading end was connected to a 10 mL syringe (Terumo, Tokyo, Japan) that was filled with PBS (dyed blue for easy observation). The test was conducted and repeated at an ever increasing flow rate until the TEVGs failed. The corresponding pressure was recoded and analyzed.

## 3. Results

**Figure 1:**
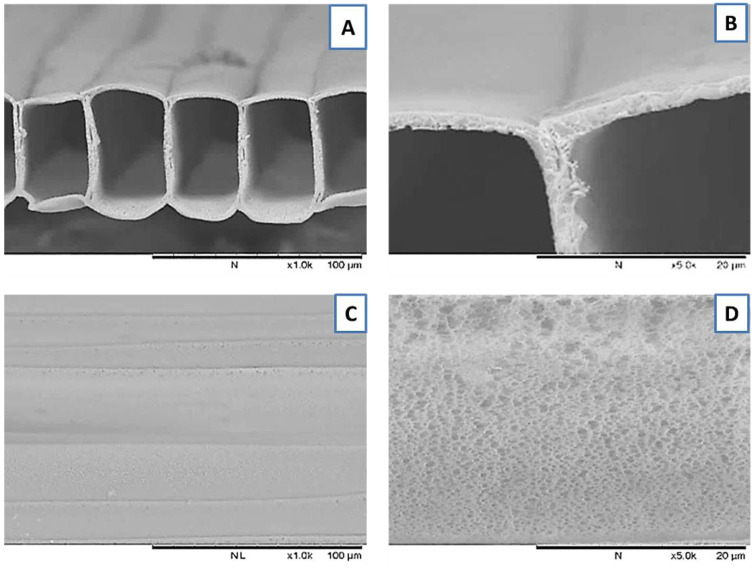
SEM images of electrospun PLGA MTAM (**A**) Transverse section; (**B**) Zoomed-in view lumen wall depicting highly porous structure; (**C**) Top view and; (**D**) Zoomed-in view of top view depicting pores.

PLGA MTAMs were successfully co-axially electrospun at a voltage range of 4.5–5.7 kV, under ambient conditions. The respective flow rates were set at 4.5 mL/h (core solution) and 5.0 mL/h (shell solution), while the collector was set at a speed of 200 rpm. The resulting PLGA MTAMs revealed highly aligned, one-to-one connected, ultra-thin, and highly porous structures that are highly similar to that of the polylactic acid (PLLA) MTAMs in our previous work (Figure 1) [37,41]. Image J analysis revealed the dimensions of the microstructures to be 57.7 ± 2.8 μm × 72.5 ± 3.6 μm (width × height), with a lumen wall thickness of 3.2 ± 0.7 μm.

**Figure 2:**
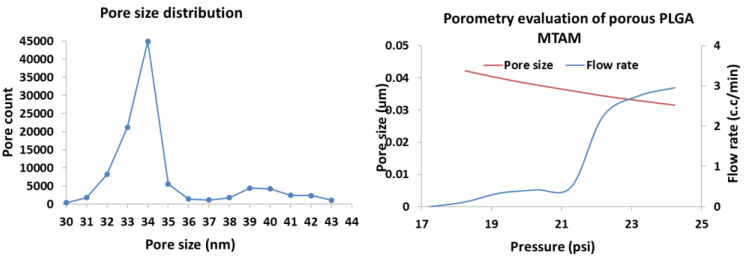
Porometry evaluation of PLGA MTAMs. The pore size distribution of the PLGA registered a mean pore size of 34 nm with a relatively narrow distribution (**left**), while the maximum burst pressure for the PLGA MTAMs was 1253.05 mmHg (**right**).

Porometry evaluation revealed the mean pore size of the PLGA MTAMs was around 34 nm. The pore size distribution was a relatively narrow with pore sizes that were mostly in the range of 32–36 nm (Figure 2). Minor quantity of pore counts, detected to be at the range of pore size of 38–43 nm, were also observed. The maximum pressure before failure of the PLGA MTAMs was registered to be 1253.05 mmHg, which was far above the pressure of most human blood vessels that has a pressure reading of 420–570 mmHg [42,43].

**Figure 3:**
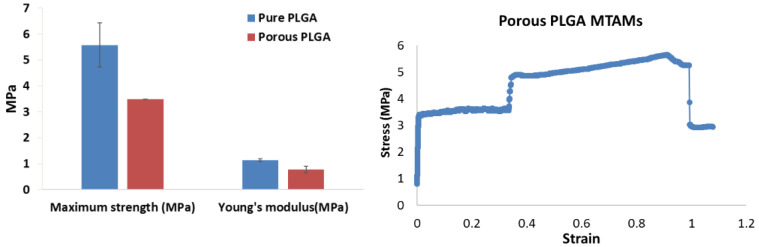
Mechanical properties (stress–strain curve) of PLGA MTAMs. The maximum tensile strength of the porous PLGA MTAMs used in the TEVG was recorded at 3.48 ± 0.06 MPa, while the Young’s Modulus registered a reading of 0.778 ± 0.13 MPa (**left**). The stress–strain (SS) curve of the porous PLGA MTAMs (**right**). A relatively sound mechanical TEVG with excellent Young’s modulus would prove to be significantly beneficial to the application.

The mechanical strength of the porous PLGA MTAM revealed a maximum tensile strength 3.48 ± 0.06 MPa and a Young’s modulus value of 0.778 ± 0.13 MPa (Figure 3). When compared to the non-porous PLGA MTAMs, these readings suggested that the presence of pores significantly reduced the overall mechanical strength, which was reasonable considering the amount of material that is subjected to stress within a specific area is less. Despite being weaker, the lower Young’s modulus suggested that the porous PLGA MTAM is a much more ductile material. Furthermore, the strain measured for the porous PLGA MTAMs seemed to suggest that the porous PLGA MTAMs was capable of being elongated by up to 1 cm before failing.

**Figure 4:**
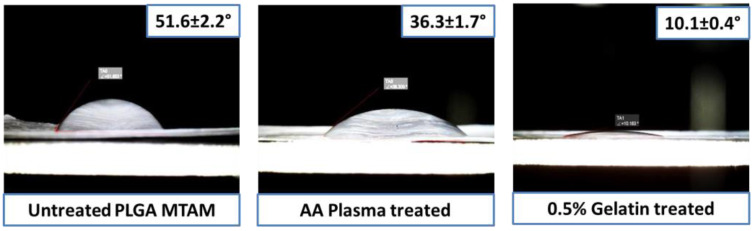
ddH_2_O contact angle of (left to right) untreated; AA plasma treated; and 0.5% treated PLGA MTAM. A significant reduction in water contact angle was recorded with the addition of surface treatment, which was followed by gelation coating.

AA plasma and 0.5% gelatin coating significantly reduced the water contact angle from 51.6 ± 2.2° to 10.1 ± 0.4°. This suggested that the combination treatment significantly improved the overall hydrophilicity of the PLGA MTAM, which was critical for the overall cell attachment [43]. A further coating by gelatin further reduced the water contact angle to 10.1 ± 0.4°, which suggested an excellent degree of hydrophilicity of the AA plasma treated and gelatin-coated PLGA MTAMs. Contact angle of samples is depicted in Figure 4.

**Figure 5:**
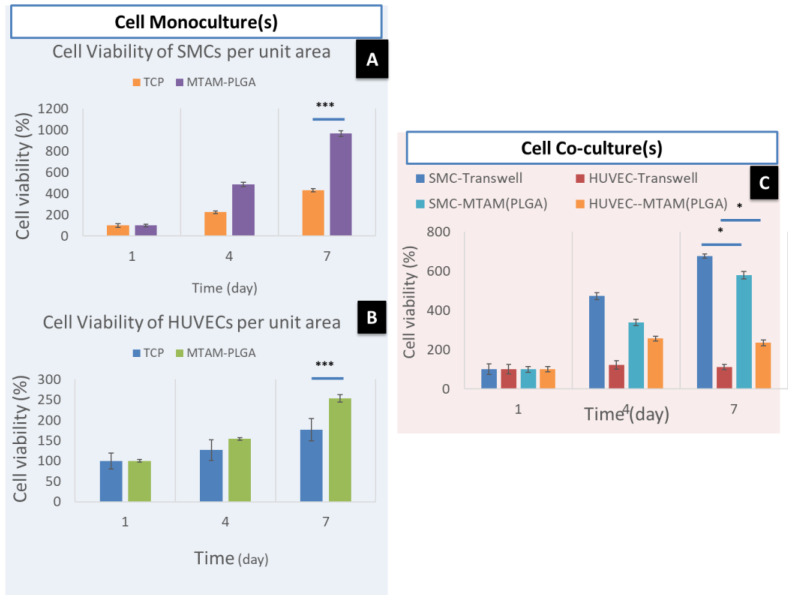
Cell viability test of SMCs and HUVECs (**A**) monoculture of SMCs; (**B**) monoculture of HUVECs; (**C**) co-culture of SMCs and HUVECs (sample size *n* = 3). * and *** mean significant.

The cell viability of the SMCs cultured in the lumens of the PLGA in a monoculture setting revealed a higher viability than the commercially available TCPs (Figure 5A). In regards to HUVECs, the cells cultured on the exterior surface of the PLGA MTAM revealed a higher viability compared to those cultured using TCPs. In the co-culture setting (Figure 5C), the proliferation of both HUVECs and SMCs revealed an increase in cell viability from day 1 to 7. However, two contrasting patterns can be observed namely, the co-culture of SMCs using Transwell system revealed a higher cellular viability compared to those co-cultured using the PLGA MTAMs and; in contrast, the HUVECs revealed a higher cellular viability when co-cultured using the PLGA MTAM compared to those co-cultured using the Transwell system.

**Figure 6:**
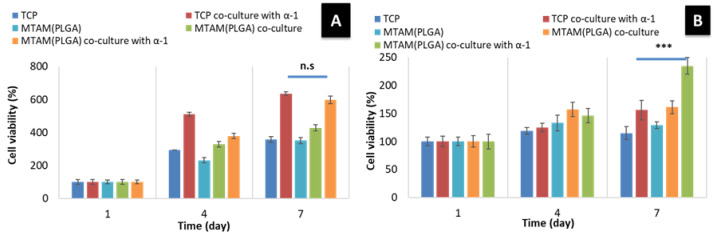
Cell viability of the co-culture of SMCs (**A**) and HUVEC (**B**) in mediums with and without Haptaglobulin α-1. In the presence of Haptaglobulin α-1, a significantly higher cell viability for both SMCs and HUVECs were observed when co-cultured using the PLGA MTAMs (sample size *n* = 3). *** means significant.

In general, the addition of Haptaglobulin α-1 (Figure 6) revealed a positive effect for both the SMCs and HUVECs in the co-culture setting. The SMCs co-cultured using the PLGA MTAMs revealed an almost similar cellular viability compared to those co-cultured using the Transwell system (Figure 6A). In contrast, the HUVECs co-cultured using the PLGA MTAMs revealed a significantly higher cellular viability compared to those co-cultured using the Transwell system (Figure 6B). This suggested that the presence of Haptaglobulin α-1 significantly improved the overall cellular proliferation and viability of the cells [44].

**Figure 7:**
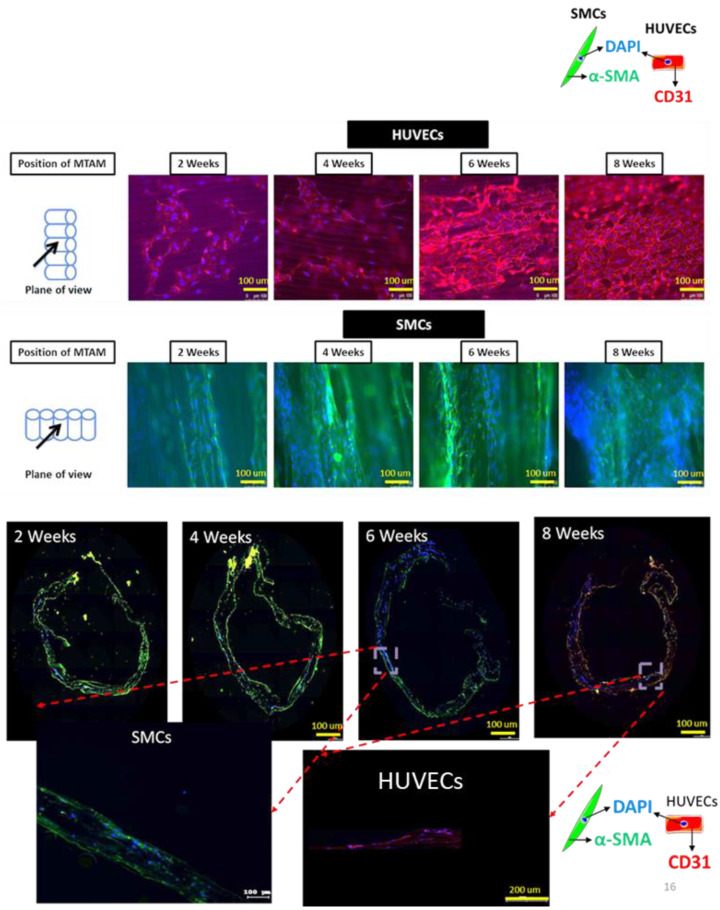
Fluorescence imaging (FM) of immunocytochemistry stained images of SMCs and HUVECs co-cultured with PLGA MTAMs (**top**), and fluorescence microscopy imaging of transverse section of 2–8-week old tissue engineered vascular grafts (**bottom**). A monolayer of HUVECs were observed by week 8 of this study (**top**, first row), while SMCs proliferated well along the direction of the lumen of the PLGA MTAMs (**top**, second row).

Fluorescence microscopy imaging (Figure 7, top) revealed that by the week 8, a monolayer of HUVECs was formed. As for the SMCs, an 8-week-old sample of the TEVG revealed a significant increase in amount of cells. More importantly, the SMCs proliferated well along the axis of the fiber alignment while maintaining its spindle-shaped morphology, which was important for the proliferation, long-term viability, and functionality of the tissue. Furthermore, the imaging of the transverse sections of the TEVG (Figure 7, bottom) revealed infiltration of SMCs into the degrading PLGA MTAM.

**Figure 8:**
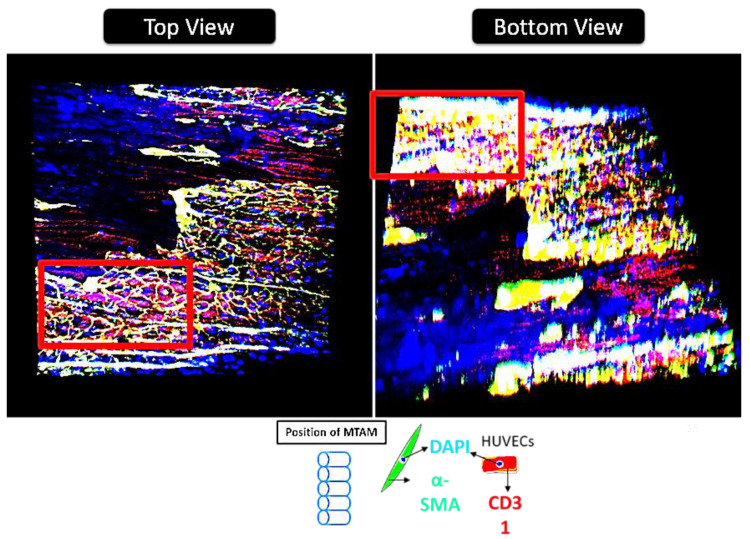
Confocal laser scanning electron microscopy (CLSM) of eight-week-old tissue engineered vascular graft (TEVG) (top view), and deconvolution microscopy (DM) of eight-week old tissue engineered vascular graft (TEVG) (bottom view). Red insert indicates indicate a similar site but a different view (i.e., top and bottom views). Red color regions indicate the presence of HUVECs, while yellow color regions are the result of overlaying of red (HUVEC) and green (SMC) colors. Blue color indicates the nucleus regions of the respective cells.

**Figure 9:**
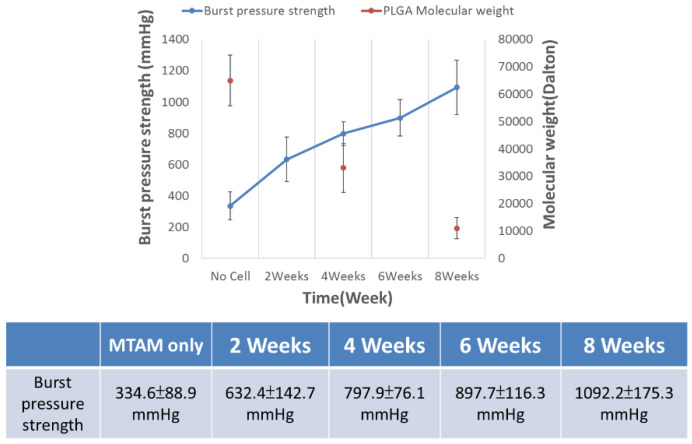
Burst pressure of 2–8 week-old tissue-engineered vascular grafts. The molecular weight of the PLGA registered a significant decrease over the entire eight weeks; on the contrary, the burst pressure increased over the entire duration of the study, ultimately achieving a burst pressure of 1092.2 ± 175.3 mmHg (sample size *n* = 3).

CLSM and DM imaging (Figure 8) further confirms the observations made earlier. These images clearly revealed the presence of a thick layer of tissue-like layers of both the SMCs and HUVECs. The presence of this thick tissue layer is extremely important for the long-term functionality of the TEVG; and this is further made evident in the increase of burst pressure over time of the TEVG (Figure 9). DM imaging (Figure 8 bottom, insert) also suggested that both the SMCs and HUVECs revealed a better proliferation and viability when co-cultured together.

Burst pressure of TEVGs increased over the entire incubation period of eight weeks (Figure 9). The decrease in the molecular weight of the PLGA MTAMs 70 kDa and which ultimately reduced to about 10 kDa was inversed to the readings obtained in the burst pressure of the TEVG, which increased from 334.6 ± 88.9 mmHg to 1092.2 ± 175.3 mmHg by the eighth week of co-culture of the SMCs and HUVECs within the PLGA MTAMs.

## 4. Discussions

PLGA MTAMs, which consisted of one-to-one connected individual ultra-thin fibers arranged in an arrayed formation, were successfully fabricated. Compared to the MTAMs of Polylactic Acid (PLLA) and Polysulfone (PSF) in our previous work, the fibers here appeared to be squared in shape, as opposed to those in our previous works [45,46,47]. The lumen walls of the PLGA MTAMs, which consisted of a homogenously porous pore with a narrow distribution of between 32–36 nm (Figure 2), suggested an excellent ability to precisely control the pore sizes through our internally developed porogen–surfactant ratio. At these sizes, the pores were sufficiently small to ensure that the cells culture within do not escape the confines of the lumen of the PLGA MTAM, while allowing nutrients, waste, and signaling factor to diffuse unhindered [38].

Furthermore, when compared to commercially available Transwell co-culture insert, the distance between the two cell types (SMCs and HUVECs in this case) were significantly shorter, thereby potentially allowing for better communication, signaling, and ultimately better viability. Such findings were confirmed in the data in Figure 6 where the SMCs registered no significant difference in viability when compared to the Transwell co-culture system, while a significantly higher viability was observed in the HUVECs cells. The findings of utilizing MTAMs as a co-culture system, which produced superior results when compared to conventional Transwell co-culture systems, were echoed in our previous work with neuron stem cells (NSCs) and astrocytes, which resulted in better viability and maturation of NSCs [47].

In another interesting property of the PLGA MTAMs, the highly aligned nature of MTAMs was extremely beneficial for the culture of cells with alignment. In this study, the SMCs proliferated very well in the direction of the fiber alignment while maintaining the critical ‘spindle shape’, which was not present in the Transwell co-culture system; they play a critical role in inducing directional proliferation, which is important for both long-term survival of SMCs and the function of the SMC tissue as a whole, as it induced the directional proliferation of the SMCs, which was critical for the promotion of DNA synthesis, overall viability, and function of SMC tissues (Figure 5 and Figure 7) [39,48,49,50,51].

Interestingly by rolling the SMCs-seeded PLGA MTAMs perpendicular to the direction of the blood flow (Figure 7 and graphical abstract), the burst pressure of the TEVG actually increased, despite the polymer graft registering significant reduction of molecular weight due to degradation (Figure 9). This can serve as indirect evidence of SMCs tissue formation; it is well known that the SMCs were responsible for providing mechanical strength in native tissues through the formation of a matrix that is only present in the tissue stage [52,53]; and in combination with the microscopy images in Figure 7 and Figure 8, which revealed formation of tissues.

By seeding the HUVECs into the inner lumen surface of the SMCs-lined, rolled PLGA MTAMs, it ultimately formed a monolayer, as seen in Figure 7. This monolayer is of utmost importance as it would eventually form the endothelium of the TEVG, which prevents the formation of thrombosis that often plagues earlier synthetic vascular grafts, lacking a functional endothelium [54,55]. Furthermore, closer examination of the monolayer of 8-week-old HUVECs potentially suggested the presence of tight junctions between adjacent cells, playing a key role in the overall success of TEVGs [56]. Such findings were made possible due to the presence of a highly interconnected 3D matrix, which was also highly porous (Figure 1B,D), promoting cellular infiltration [57]. It was also observable that the amount of SMCs infiltration seemed to increased overtime, as the degrading PLGA MTAM resulted in an overall increase in porosity, which, in turn, allowed for better cellular infiltration [57,58].

Notwithstanding, it was also critical to note that the selection of material for the fabrication of MTAMs was of utmost importance, as we were unable to obtain a similar monolayer when utilizing PLLA MTAMs (data not shown). Without a doubt, the PLGA material, that was previously used in TEVGs in works by other groups [59,60,61]; the combination of the nanoporous surface and the AA plasma treatment, which was followed by gelatin treatment that increased the hydrophilicity of the surface of the PLGA MTAMs significantly contributed to the improved viability of both the SMCs and HUVECs through the enhanced protein adhesion [62,63]. The enhanced protein adsorption in turn affected the cellular attachment and ultimately, the proliferation, and long-term viability of the cells [64]. Furthermore, the PLGA MTAMs provided a 3D scaffold substrate, which more closely mimicked the native conditions of these cells [65]. The use of gelatin treatment, which resulted in an improved outcome for vascular-related cells, were echoed by the works of other groups [66,67].

With the addition of Haptaglobulin α-1, the SMCs co-cultured using the PLGA MTAMs revealed an almost similar cellular viability compared to those co-cultured using the Transwell system (Figure 6). In contrast, the HUVECs co-cultured using the PLGA MTAMs revealed a significantly higher cellular viability compared to those co-cultured using the Transwell system (Figure 6). This suggested that the presence of Haptaglobulin α-1 significantly improved the overall cellular proliferation and viability of cells [44]. One possible explanation for the significant improvement of the co-culture of SMCs and HUVECs when Haptaglobulin α-1 was administered lies in the absence of fibroblast in the co-culture equation. In the native blood vessels, Haptaglobulin α-1 are secreted by fibroblast within the immediate injured area of the arteries, and these Haptaglobulin α-1 are responsible for the breakdown of gelatins in the process of arterial restructuring, resulting in the formation of gelatin matrix complexes that promotes cellular migration and tissue formation [68].

Mechanically speaking, PLGA MTAMs are relatively ductile material (Figure 3) and with a maximum burst pressure of 1253.05 mmHg, which was superior to most native human microvascular with readings of 420–570 mmHg [42]. Furthermore, the relatively low Young’s Modulus, which registered a reading of 0.778 ± 0.13 MPa, was within the readings of the native human aorta [69]. The relative low Young’s Modulus of the PLGA MTAMs was actually beneficial to the culture of endothelial cells and might have contributed to the excellent output in both the HUVECs and SMCs in this study. The reason for this deduction was because it was known that a low Young’s modulus was beneficial for endothelial cell culture in biomaterial substrate as it was observed that the increase in the Young’s modulus resulted in the inhibition of cellular proliferation, which was suggested for the result of reduced expression extracellular matrix (ECM) proteins namely; fibronectin, collagen IV (alpha 1; α1), collagen IV (alpha 1; α5), and Heparin Sulfate Proteoglycan (HSPGs-perlecan and biglycan), which ultimately affects tissue formation for the respective endothelial cells [70].

By the eighth week, the resulting TEVGs, which have only about 18% of original molecular weight of the PLGA scaffold left, and with the majority burst pressure sustained by the newly formed SMCs tissues resulted in the TEVG being capable of sustaining pressures of up to 1092.2 ± 175.3 mmHg. Despite not being sufficient for major arteries, which requires burst pressures of upwards of 2000 mmHg [71,72], the burst pressure that the TEVG sustained was sufficiently high for blood vessels such as human umbilical artery 969.66 ± 154.42 mmHg and for use in small diameter vascular applications, which generally have a burst pressure between 60–300 mmHg [72].

## 5. Conclusions

In conclusion, TEVG based on the co-culture of the SMCs and HUVECs with the electrospun PLGA MTAMs revealed good cellular proliferation, degradation rate, and sufficient burst pressure for applications such as small-diameter, tissue-engineered vascular grafts. Potentially, with further research and development, this scaffold can be utilized for coronary artery bypass surgeries.

## Data Availability

Not applicable.
